# The ProVIDe study: the impact of protein intravenous nutrition on development in extremely low birthweight babies

**DOI:** 10.1186/s12887-015-0411-y

**Published:** 2015-08-26

**Authors:** Frank H. Bloomfield, Caroline A. Crowther, Jane E. Harding, Cathryn A. Conlon, Yannan Jiang, Barbara E. Cormack

**Affiliations:** Liggins Institute, The University of Auckland, Auckland, New Zealand; Newborn Services, Auckland City Hospital, Auckland, New Zealand; Gravida: National Centre for Growth and Development, Auckland, New Zealand; Auckland Academic Health Alliance, Auckland, New Zealand; Department of Paediatrics: Child and Youth Health, The University of Auckland, Auckland, New Zealand; The Robinson Institute, The University of Adelaide, Adelaide, Australia; School of Food and Nutrition, College of Health, Massey University, Auckland, New Zealand; Department of Statistics, Faculty of Science, The University of Auckland, Auckland, New Zealand

**Keywords:** Preterm infant, Nutrition, Growth, Protein, Neurodevelopment

## Abstract

**Background:**

Preterm birth and very small size at birth have long-term effects on neurodevelopment and growth. A relatively small percentage of extremely low birthweight babies suffer from severe neurological disability; however, up to 50 % experience some neurodevelopmental or learning disability in childhood. Current international consensus is that increased protein intake in the neonatal period improves both neurodevelopment and growth, but the quantum of protein required is not known. This trial aims to assess whether providing an extra 1 to 2 g.kg^-1^.d^-1^ protein in the first 5 days after birth will improve neurodevelopmental outcomes and growth in extremely low birthweight babies.

**Methods/Design:**

The ProVIDe study is a multicentre, two-arm, double-blind, parallel, randomised, controlled trial. In addition to standard intravenous nutrition, 430 babies with a birthweight of less than 1000 g who have an umbilical arterial line *in situ* will be randomised in 1:1 ratio to receive either an amino acid solution (TrophAmine®) or placebo (saline) administered through the umbilical arterial catheter for the first 5 days. Exclusion criteria are admission to neonatal intensive care more than 24 h after birth; multiple births of more than 2 babies; known chromosomal or genetic abnormality, or congenital disorder affecting growth; inborn error of metabolism, and in danger of imminent death.

*Primary outcome:* Survival free from neurodevelopmental disability at 2 years’ corrected age, where neurodevelopmental disability is defined as cerebral palsy, blindness, deafness, developmental delay (standardised score more than 1 SD below the mean on the cognitive, language or motor subscales of the Bayley Scales of Infant Development Edition 3), or Gross Motor Function Classification System score ≥1.

*Secondary outcomes:* Growth, from birth to 36 weeks’ corrected gestational age, at neonatal intensive care discharge and at 2 years’ corrected age; body composition at 36 to 42 weeks’ corrected postmenstrual age and at 2 years’ corrected age; neonatal morbidity, including length of stay; nutritional intake.

**Discussion:**

This trial will provide the first direct evidence of the effects of giving preterm babies a higher intake of intravenous protein in the first week after birth on neurodevelopmental outcomes at 2 years corrected age.

**Trial registration:**

Australian New Zealand Clinical Trials Registry: ACTRN12612001084875.

## Background

Advances in neonatal intensive care mean that the majority of preterm babies, even at the extremes of gestation, now survive infancy, childhood and adolescence [1]. The ultimate goal for neonatal care is optimal growth, neurodevelopment and long-term health for survivors [2]. A relatively small percentage of preterm babies suffer from severe neurological impairment; however, up to 50 % of extremely low birthweight (ELBW; birthweight <1000 g) babies experience some neurodevelopmental or learning impairment in childhood [3]. Current international consensus is that increased protein intake in the neonatal period improves neurodevelopment, growth, and body composition, but the quantum of protein required is not known. Therefore, it is now crucial that fundamental elements of neonatal nutrition care, such as the protein intake required for optimal growth and how this influences neurodevelopment and long-term health for preterm babies, are addressed.

The fastest rate of postnatal growth in the human lifecycle is (potentially) that of a preterm baby between 23 and 27 weeks’ gestation when weight gain is approximately 21 g.kg^-1^.d^-1^ [4]. However, faltering growth at the equivalent gestational age is common in preterm babies [5, 6], due to the difficulties in providing nutrition sufficient to support this rate of growth [6, 7]. Preterm babies frequently suffer both fetal and postnatal growth restriction leading to a different pattern of postnatal weight gain and body composition, including lower length at term-equivalent age, lower lean muscle mass, and higher total body fat mass [6, 8–10]. Each of these patterns of growth is associated with an increased risk of adult disease [11–13] and both fetal growth restriction and postnatal faltering growth also are associated with adverse neurodevelopmental outcome [14, 15].

Suboptimal nutrition likely contributes to both the faltering postnatal growth and impaired neurodevelopmental outcomes that are seen commonly in ELBW babies [16, 17]. In preterm babies, length and head growth appear to be affected early, in contrast to the classic faltering growth of poorly nourished term infants, with Z-scores falling by 1–2 standard deviations for all measures of growth during the first month of postnatal life [18, 19]. This early effect on head growth likely reflects the extremely rapid brain growth that occurs at the gestational ages ELBW babies are receiving suboptimal nutrition, as head circumference correlates with brain volume [20]. Volumetric magnetic resonance imaging (MRI) analyses suggest that cerebellar surface area increases 30-fold in the last 16 weeks of pregnancy and cerebral cortical volume increases four-fold from 28 to 40 weeks’ gestational age [21–23]. This very rapid development may also result in greater vulnerability to damage from less than optimal nutrition. Indeed, in adolescents born preterm, performance in final school exams and intelligence quotient (IQ) score are best predicted by the total volume of white matter in the brain detected on MRI scans, irrespective of the presence or severity of other brain abnormalities [24], with 70 % of the variance in IQ explained by total white matter volume and the cross-sectional area of the corpus callosum [24].

Thus, a key modifiable factor for improving neurodevelopmental outcome in preterm babies is improving growth, especially head growth, by optimising nutritional intake in the early postnatal period. Retrospective data suggest that, across a range of protein intakes that were below international recommendations, each 1 g.kg^-1^.d^-1^ increase in protein intake in the first week after birth in ELBW babies improves Mental Development Index scores by 8 points on a Bayley II assessment at 18 months [25]. However, not all data are consistent in this regard [26], few data are from robust randomised controlled trials and even fewer data are from studies in which protein and other nutrient intakes met international recommendations.

We previously have reported that babies with a birthweight of <1200 g failed to meet consensus recommended intravenous nutrient intakes [7, 27–29], consistent with international experience [30, 31], and that this was particularly common in ELBW babies. Close adherence to nutritional guidelines designed to increase nutritional intakes to match international recommendations does result in both increased protein intakes and significantly better growth for weight, length and head circumference, although the benefit for length growth was less than that for weight and head circumference [18]. However, despite these improvements, growth rates are still less than intrauterine growth velocity at comparable gestational ages, the currently accepted recommended goal for ELBW babies [32]. This may be because protein intake in the first week after birth remains less than estimated placental protein uptake *in utero* between 23 and 27 weeks’ gestational age (3.6 to 4.8 g.kg^-1^.d^-1^) [33] and is, therefore, still insufficient to support *in utero* protein accretion rates. Indeed, better linear growth is associated with higher protein intakes in the first week after birth [19]. We, therefore, hypothesise that a higher protein intake during the first week after birth will improve neurodevelopmental outcomes at age 2 years’ corrected age.

In clinical practice, recommended protein intakes are difficult to achieve due to the low fluid volumes administered to ELBW babies during the first week after birth and their need for other infusions containing non-nutritive fluids such as drugs. We propose to utilise the umbilical artery catheter (UAC) to deliver additional protein, substituting the non-nutritive fluids usually given via the UAC with an amino acid solution. This research will fill an urgent need for data from randomised controlled trials investigating the effect of early nutritional practices on clinically important outcomes in these most vulnerable babies.

### Hypothesis

The primary hypothesis is that for ELBW babies, an extra 1 g.d^-1^ of protein in the first 5 days after birth will improve survival free from any neurodevelopmental disability at 2 years’ corrected age. The secondary hypotheses are that increased protein intake in the first week after birth has benefits relating to neonatal morbidity, length of neonatal intensive care unit (NICU) stay, growth and body composition.

### Aims and objectives of this trial

We propose replacing the non-nutritional fluids (saline) that currently are administered via the UAC with an intravenous amino acid solution and will assess the effect of this intervention through a randomised, placebo controlled trial. The aim is to determine whether an additional 1 to 2 g.kg^-1^.d^-1^ protein (amino acid solution) via the UAC starting within 24 h of birth and continued for 5 days will (i) improve survival free of neurodisability at age 2 years’ corrected; (ii) improve body composition, and (iii) prevent faltering growth at NICU discharge.

Specific aims are to determine the effect of the intervention on:

- Survival free of neurodisability at 2 years’ corrected age
- Growth, from birth to 36 weeks’ postmenstrual age, at NICU discharge and at 2 years’ corrected age.
- Body composition, at 36 weeks’ postmenstrual age measured by air displacement plethysmography, and at 2 years’ corrected age
- Neonatal morbidity, including length of stay
- Nutritional intake

## Methods/Design

### Trial design

Multicentre, double-blind, two-arm, parallel, randomised, controlled trial.

### Ethical approval

The Northern B Health and Disability Ethics Committee has given ethical approval for this study (No 13/NTB/84), and each participating site has institutional approval through local institutional review processes. The New Zealand National Screening Unit has given approval for access to the Newborn Metabolic Screening data.

### Inclusion criteria

Babies with a birthweight of less than 1000 g who have a UAC *in situ* in an acceptable position.

### Exclusion criteria

Babies admitted to neonatal intensive care more than 24 h after birth; multiple births of more than 2 babies; known chromosomal or genetic abnormality, or congenital disorder affecting growth; inborn error of metabolism; in danger of imminent death.

### Trial entry and randomisation

Parents will be given a written information sheet about the study antenatally (where possible), which will be reviewed with them by a member of the study team. If birth occurs without time for this to happen, information will be given to the parents as soon as is feasible after birth and reviewed with them by a member of the study team. Within 24 h of birth and once a UAC has been placed, eligible babies will be randomised to either the placebo group or the intervention group. Babies for whom it was not possible to obtain consent antenatally will be enrolled based on a waiver of consent (approved by the ethics committee) with a requirement that informed, written consent is obtained within 24 h. If consent is not obtained within 24 h of randomisation, the baby will be withdrawn from the study.

### Study groups and management

Babies in the placebo group will receive 0.45 % saline through the UAC (standard therapy). Babies in the intervention group will receive an amino acid solution (8.5 % TrophAmine®, B Braun Medical, Irvine, USA) through the UAC giving an extra 1 g.d^-1^ of protein above standard intravenous nutrition. Babies in both groups will receive routine neonatal care including intravenous and enteral nutrition according to each centre’s practice.

### Randomisation

Within 24 h of birth and once a UAC has been placed in an acceptable position, babies will be enrolled by medical and research staff and randomly allocated in a 1:1 ratio to treatment and placebo groups via a web-based interface maintained and concealed by an independent database controller. Twins will be randomised as separate babies. Randomisation will be stratified for recruitment centre (each centre has different nutrition practices), sex and appropriate-for-gestational-age/small-for-gestational-age status (these variables influence growth and body composition), using blocked randomisation with variable block sizes.

### Blinding

Subjects and their families, clinical staff, investigators and assessors at follow up appointments will be blinded to treatment allocation throughout the entire study. Intervention and placebo fluids will be made by an independent contractor and will be identical in appearance and identified only by a randomly generated numerical identifier. Composition of the fluid in each bag will be known only by the independent database controller. Unblinding should occur only in exceptional circumstances when knowledge of the actual treatment is absolutely essential for further management of the patient. If unbinding is deemed to be necessary by a neonatologist, the neonatologist is encouraged to discuss this with the overall responsible investigator (FHB). The actual allocation will not be disclosed to parents and/or other study personnel. Any code break that occurs will be reported to the principal investigator and Steering Group without identifying treatment allocation.

### Intervention

All babies will receive nutrition according to individual neonatal intensive care unit practices. In addition, babies will be randomised to one of two groups:

Treatment group: Babies will receive an infusion of 8.5 % TrophAmine® at 0.5 ml.h^−1^ via the UAC, providing 1 g protein in 12 ml or 1–2 g.kg^−1^.d^−1^ additional protein, depending on birthweight. Placebo group: Babies will receive an infusion of 0.45 % saline at 0.5 ml.h^−1^ via a UAC (current standard practice). Both solutions will contain heparin 0.5 iU.ml^-1^. The intervention will continue for 120 h.

#### Blood sample analysis

Routine biochemical monitoring will take place in accordance with local guidelines. Concentrations of potassium, pH, bicarbonate, base excess and lactate will be recorded daily. In addition, blood samples will be collected on day 1 (24 h after UAC infusion starts) and day 5 (after the UAC study fluids cease) for the measurement of serum concentrations of urea, albumin, total protein, globulin, calcium and phosphate concentrations on days 1 and 5, and ammonia concentration on day 5. Study blood results will not be revealed to attending physicians.

#### Newborn metabolic screening

Blood spots taken for routine Newborn Metabolic Screening will be taken immediately before and after the 5-day intervention period. Consent has been granted by the New Zealand National Screening Unit for access to the Newborn Metabolic Screening data, including amino acid and acyl carnitine concentrations.

#### Monitoring of nutritional intake

Total enteral and intravenous intakes will be recorded daily until day 28. Mean daily protein, energy and other nutrient intakes will be calculated based on actual intakes. Full enteral feeds will be defined as the day when no further intravenous nutrition is given or 150 ml.kg^-1^d^-^^1^ enteral feeds is reached. Energy and protein intakes will be calculated using preterm transitional breast-milk composition for the first 2 weeks (65 kcal and 1.5 g protein.100 ml^−1^) and mature breastmilk composition thereafter (72 kcal and 1.2 g protein.100 ml^−1^) [34–38].

#### Participant withdrawal

Development of significant renal impairment (serum creatinine > 130 μmol.l^−1^) or diagnosis of an inborn error of metabolism requiring prescribed protein intakes will result in the intervention being discontinued. Participants will be withdrawn from the trial at parental request or if the attending physician determines that this is essential for the baby’s care. Consent will be requested to continue to use routinely collected data for study purposes.

### Study outcomes

#### Primary outcome

The primary outcome will be survival free from any neurodevelopmental disability at 2 years’ corrected age. Assessment will include the Bayley Scales of Infant Development Edition 3 (Bayley III), neurodevelopment, growth and body composition. Cerebral palsy (loss of motor function and abnormalities of muscle tone and power) and other impairment outcomes will be assessed according to previously reported criteria [39]. The severity of gross motor function will be classified using the Gross Motor Function Classification System [40]. Children with severe developmental delay who are unable to complete the psychological assessment will be given a standardised score of - 4 SD. Children will be considered blind if visual acuity in both eyes is worse than 6/60 and deaf if their hearing loss is sufficient to require hearing aid(s), or worse.

Children will be considered to have a neurodevelopmental disability if they have cerebral palsy, a gross motor classification score ≥1 [40], blindness, deafness or developmental delay, defined as a standardised score for cognitive, motor or language scales more than 1 SD below the mean [41]. The neurodevelopmental disabilities imposed by the various neurodevelopmental impairments will be classified as severe, moderate or mild [39].

#### Secondary outcomes

##### Growth

Weight, length and head circumference will be measured at birth, 28 days, 8 and 36 weeks’ postmenstrual age, and at discharge. Weight, height and head circumference will be measured at 2 years’ corrected age. All measurements will be by trained staff using validated, repeatable methods [42, 43]. All growth data Z-scores will be calculated individually for each baby using appropriate normative data. Growth velocity (GV) will be calculated using: GV = [1000 × ln(Wn/W1)]/(Dn ‐ D1) where W = weight (g) and D = day after birth on days n (end of time interval) and 1 (beginning of time interval) [44].

##### Body composition

Body composition, where possible, will be measured by air displacement plethysmography at 36 to 42 weeks’ post-menstrual age and at 2 years’ corrected age.

##### Neonatal outcomes

The presence of morbidities at 36 weeks’ post-menstrual age, including intraventricular haemorrhage (IVH), severe IVH (Grade 3 or 4 defined using the grading system from Papile et al. [45]), periventricular leukomalacia, chronic lung disease (need for oxygen at 36 weeks’ post-menstrual age or 28 days after birth if born after 32 weeks’ gestation), retinopathy of prematurity grades 3 and 4 as per the International Classification of Retinopathy of Prematurity [46], necrotising enterocolitis (defined as Bell’s stage 2 or higher [47]), patent ductus arteriosus diagnosed by echocardiography needing treatment, length of NICU stay and late-onset sepsis (beyond 7 days after birth and defined as a positive bacterial culture in cerebrospinal fluid, urine or blood with clinical signs of infection and with antibiotics for 5 or more days with the intention of treating an infection, or treatment for a shorter period if the patient died. If after 10 days of appropriate antibiotic therapy there was demonstration of sterile culture and then the same organism was cultured or if a different organism was cultured from a subsequent culture, this will be considered an additional episode).

##### Nutritional intake

Intravenous and enteral nutritional intake until 28 days (fluid, energy, protein, fat, carbohydrate, vitamins and minerals). Feed type and nutritional supplementation at NICU discharge.

#### Statistical considerations

##### Sample size

A total of 430 babies (*n* = 215 in each group) will provide 85 % power at a 5 % level of significance (two-sided), to detect an absolute difference of 15 % in survival free of disability between the two groups at 2 years’ corrected age. The sample size has taken into account a 10 % loss to follow up rate and a disability rate in the control group of 50 %.

##### Statistical analyses

Statistical analyses will be performed on an intention-to-treat basis. Missing outcome data will not be imputed in the primary analysis, as the key assumption of missing at random is not likely to hold in the analysis population. Sensitivity analyses will be conducted, however, using multiple imputations method to explore the potential impact of missing data on outcomes. The characteristics of those participants with missing data will be compared between two treatment groups. Per protocol analysis will be performed for those who received at least 80 % of the intended treatment. Sub-group analysis will be conducted on the treatment outcomes in relation to total protein received and to the extra study dose received. Statistical tests will be two-sided at a 5 % significance level. Twins will be randomised as separate babies, with the non-independence of these pairs taken into account during analysis.

Baseline characteristics of all randomised babies and their mothers will be summarised for each group as well as overall using descriptive statistics. Continuous variables will be reported as numbers of observed and missing values, mean, standard deviation, median and range. Categorical variables will be described as frequencies and percentages. The primary outcome, survival free of disability, will be first presented as unadjusted relative risk (RR) with 95 % confidence interval (CI). Generalised linear regression models appropriate to continuous and categorical outcomes will be used to evaluate the treatment difference between two groups, adjusting for stratification variables and other baseline confounders that are closely associated with the outcomes (e.g. birth weight, sex and treatment with antenatal steroids). Adjusted RRs and 95 % CIs will be estimated using a log link. Analyses of growth outcomes will be performed following completion of NICU discharge assessments but will not be revealed to assessors of the Bayley III at 2 years’ corrected age.

##### Data monitoring and other quality control measures

A Trial Steering Committee has been formed to monitor the conduct of the study. The terms of reference were agreed at the first meeting (before the trial began). Trial procedures will be in accordance with the CONSORT guidelines [48, 49]. The Trial Steering Committee is responsible for advising investigators regarding trial continuation or cessation should issues of futility or safety arise, and meet within a month of all Data Monitoring Committee meetings to consider their recommendations.

The terms of reference for an independent Data Monitoring Committee were agreed at the first meeting. An independent Safety Monitoring Committee has also been formed. During the period of recruitment, aggregated summaries of death or other serious adverse event that the local investigator believes should be referred will be supplied, in strict confidence, to the Safety Monitoring Committee. The Safety Monitoring Committee will review individual reports of adverse events. Group allocation will not be revealed to the Safety Monitoring Committee or the investigators. Should the Safety Monitoring Committee rule that the intervention may have impacted on the adverse outcome, this will be immediately reported to the chair of the Trial Steering Committee. The Steering Committee will decide on the actions to be taken.

## Discussion

This multicentre randomised controlled clinical trial aims to assess whether a higher protein intake in the first 5 days after birth will improve survival free of disability in extremely low birthweight babies. Until data from large, well-designed randomised trials are available to assess the effects of earlier higher intravenous protein intakes it is difficult to develop meaningful, evidence-based nutrition guidelines. A conclusive outcome will provide important, reliable evidence of great relevance for the nutritional management all preterm babies <1000 g at birth in settings where intensive care and intravenous nutrition are provided, because it involves administering additional protein through the simple substitution of one fluid for another using an existing commercial amino acid solution.

This intervention has the potential to improve significantly both short and long-term health outcomes for preterm babies and, therefore, to reduce health costs for these children as they reach adolescence and adulthood. If successful this simple, readily available and low cost intervention is likely to result in a rapid change in international practice, benefiting preterm babies worldwide.

## Abbreviations

ELBW: Extremely low birthweight
UAC: Umbilical arterial catheter
Bayley III: Bayley Scales of Infant Development Third Edition Bayley III
NICU: Neonatal intensive care unit
Z-score: Standard deviation score
MRI: Volumetric magnetic resonance imaging
IQ: Intelligence quotient
CI: Confidence interval
